# A formula for survival in surgery

**DOI:** 10.1186/s13037-023-00362-z

**Published:** 2023-05-27

**Authors:** Kjetil Søreide

**Affiliations:** 1 Department of Gastrointestinal Surgery, Stavanger University Hospital, Stavanger, Norway; 2 SAFER Surgery, Stavanger University Hospital, Stavanger, Norway; 3 Department of Clinical Medicine, University of Bergen, Bergen, Norway

**Keywords:** Surgical education, Patient safety, Surgical outcomes, Research, Surgical science, Implementation

## Introduction

Surgeons continuously work to ensure that their patients receive safe, timely and high-quality care, often in challenging circumstances. The strive for excellence may often manifest itself in the surgeons’ obsessive pursuit for technical perfection with focus on minute details of the procedure itself. Despite the efforts, adverse events still occur, leading to suboptimal results for patients and sometimes to fatal outcomes. A high global variation in post-operative mortality exist after both elective and emergency surgery [1, 2], across clinical pathways and for specific procedures [3].

Most elective surgical procedures have a very low risk for death, mortality as an outcome metric is most reliably used in situations or for conditions with high stakes and high risks involved. Patients present with emergency conditions for which surgeons are expected to lead a team in diagnostic work up to make the right decisions for best management. For emergency surgery, the risk of death increases several-fold compared to similar elective surgery (e.g. elective abdominal aortic aneurysm repair compared to ruptured abdominal aortic aneurysm), with persistent and in part unexplained variation in the mortality reported across and within health care systems.

Even though surgical technical details are important and not to be neglected, the outcome after surgery depends just as much on other attributes to surgery as the mere technical performance of the procedure. Thus, a hypothetical framework to build a “formula for survival in surgery” is presented to allow for dedicated quality-improvement on safety elements to surgery with the aim to improve survival.

## The theory behind the ‘formula for survival’

Two decades ago, an international working group in the field of resuscitation introduced a hypothetical formula for survival in cardiopulmonary resuscitation that included the interactive elements of *science*, *education* and *local implementation* to determine *survival* of cardiopulmonary resuscitation [4]. The concept has since been refined across several areas of resuscitation on a global scale, including the Helping Babies Survive and Helping Mothers Survive programs [5].

## A formula for survival in surgery

As follows from the above, a generic “formula for survival in surgery” is presented as it may be applied to surgery. In the original paper proposing the formula for survival [4], the focus was on cardiopulmonary resuscitation, and the authors proposed a theoretical model of putative effect from each of the multiplicands (*science* x *education* x *local implementation* = *survival*). The original theory highlighted how each component needed to be maximized to achieve optimal survival in cardiac arrest.

**Fig. 1:**
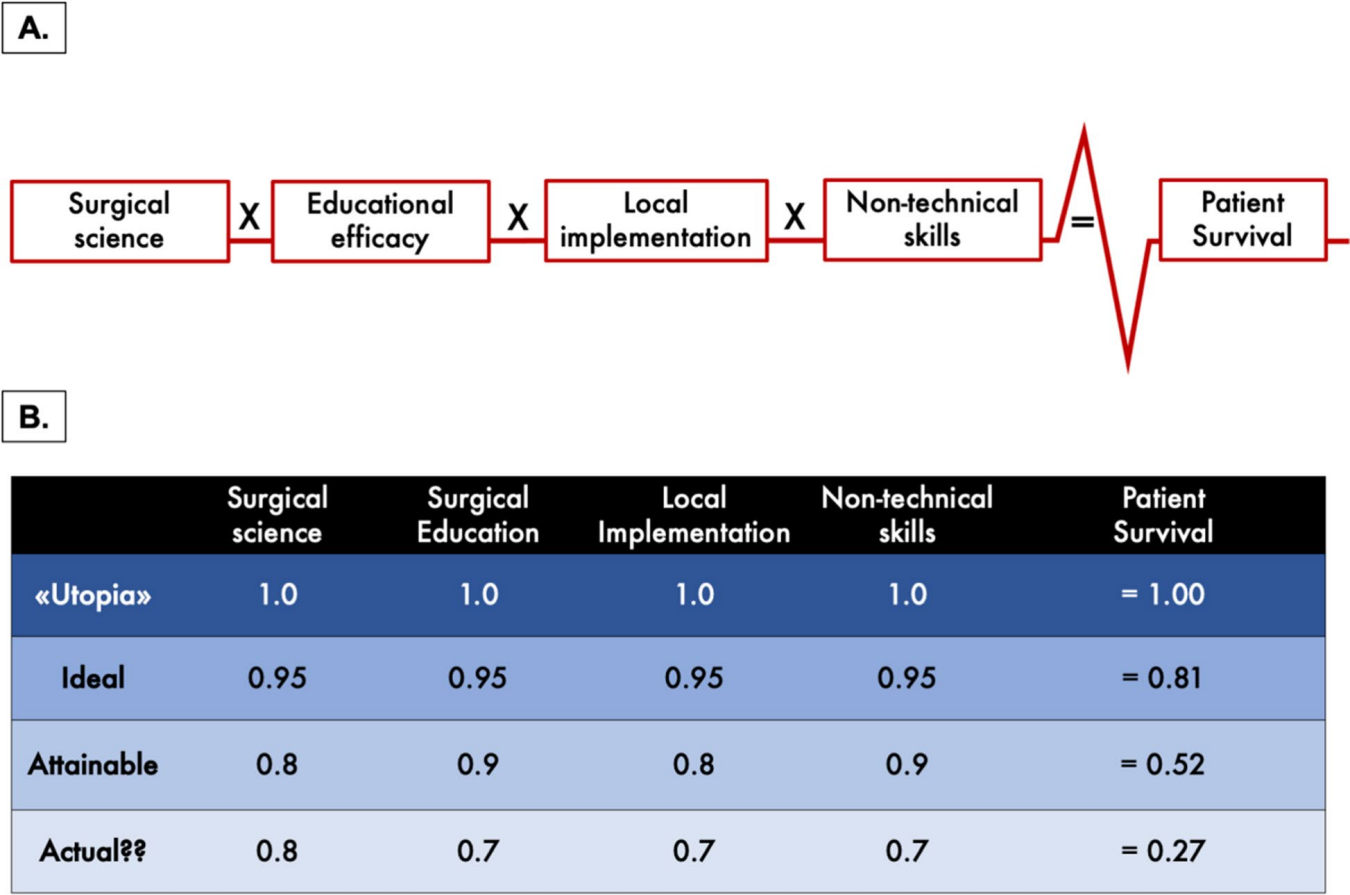
A formula for survival in surgery. **A** The components of the formula for survival in surgery. **B** A table with hypothetical settings of the formula for survival in surgery, from an ‘utopian’ model to what is potentially achievable

Accordingly, the generic ‘formula for survival in surgery’ is based on *surgical science*, *surgical education*, and *local implementation*. In addition, the added value of *non-operative technical skills* represents a novel contributing factor to the formula (Fig. 1A), and is not previously reported. Notably, the recognition of non-technical skills has become a pillar to understand and improve surgical interactive care and is increasingly understood as a core part of surgeons’ provision of an efficient and safe patient journey.

**Fig. 2:**
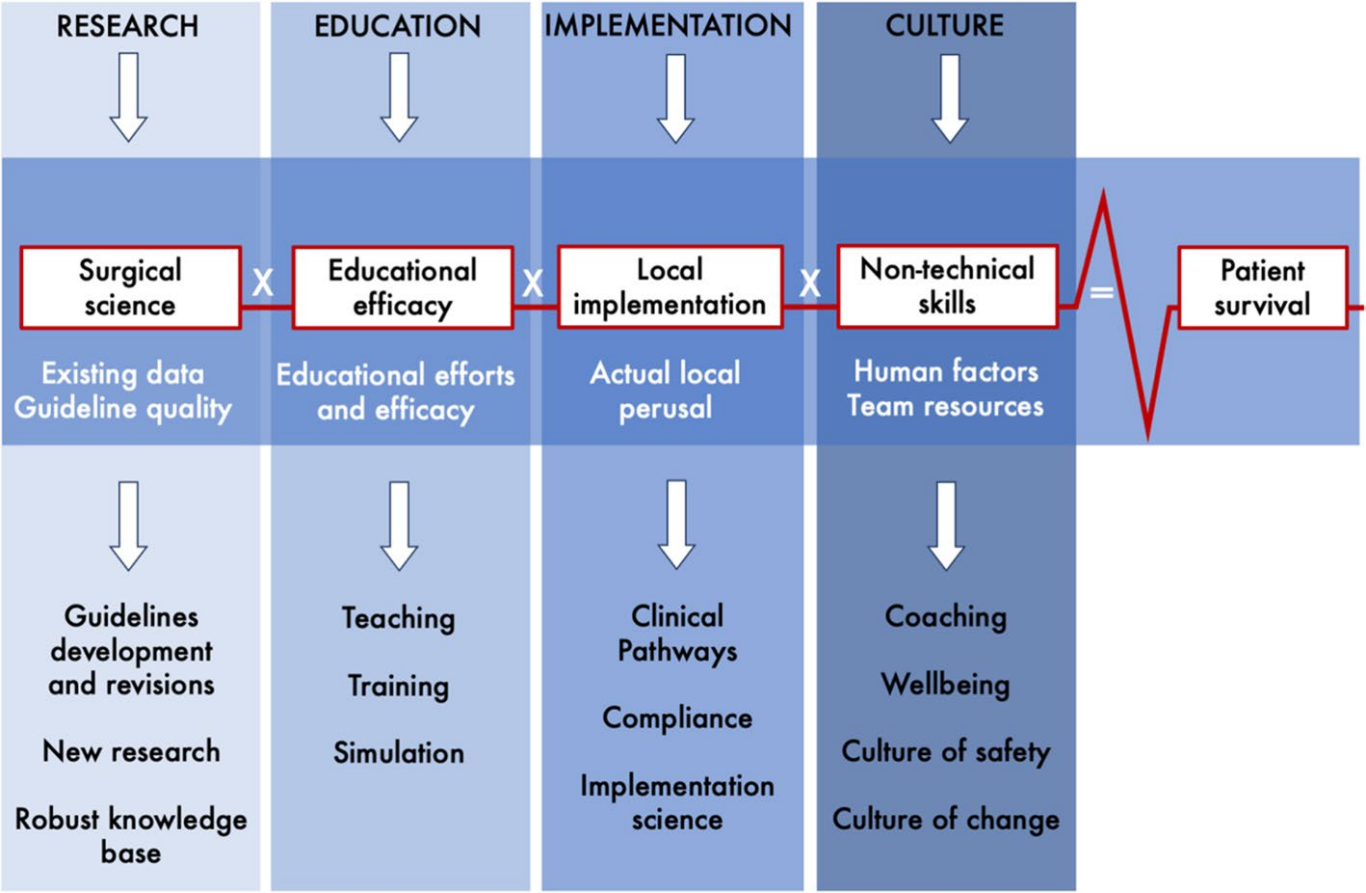
A formula for survival in surgery framework for improved quality and safety. The model is generic and should be tailored to fit specific needs for a given surgical condition or surgical specialism

In an “utopian” scenario (Fig. 1B) each of the factors are designated a perfect arbitrary value of 1 with the resultant outcome (1 × 1 × 1 × 1 = 1) as being ‘perfect’. One should not misinterpret this as achieving “100% survival”, as there are likely biological limits to any given condition. As such, the formula and its arbitrary values are not exact measures and is not a mathematical formula that depends on rigorous measures of scientific metrics. Rather, the formula serves as an educational tool to illustrate the importance of a series of elements and how they each interconnect to influence on an eventual outcome. Thus, the values presented should not be interpreted as exact, but rather as a relative value unit to gauge an intended and wanted impact on outcomes. The arbitrary values propose a measure by which to assess how far we are from the optimal setting of delivering best possible care for a given condition or disease. Each component has an additive effect towards the outcome (Fig. 2), but also represents a weak link if not attended to in the chain of care.

## Surgical science

Best practice should derive from best evidence, based on robust data obtained through high-quality research methods that address clinically relevant questions. Unfortunately, surgical research has been dubbed a ‘comic opera’ for its low-quality and often retrospective, single-centered (even single-surgeon) focus in the past [6]. However, much has been improved in surgical research, with efforts towards research collaboration, increase in volume and quality of trials and use of novel trial designs. Nonetheless, the amount of research activity (or, rather the lack thereof) is correlated to higher cancer-related mortality [7]. Fewer than 1% of cancer patients are enrolled in clinical trials [8], and surgery is described as one of the factors associated with poor trial enrolment or trails failing to complete. It may be that the research literacy among surgeons outside the traditional academic centers may be low and that the nature of surgery as a clinical discipline has not fostered similar interest to academic work as for non-operative specialisms. Thus, one can envision that there is much to gain in “surgical science” before reaching the utopian score of 1. Notably, several guidelines in surgery have clinical recommendations that are based on very low grade of evidence. Still, it may constitute what is best practice based on best obtainable knowledge. Also, the quality of research synthesis of existing data seems to suffer from high variation [9]. Hence, many “impossible” trials not done, or “difficult” research projects not performed today, may eventually contribute to higher value, better content, and more robust knowledge when championed by the next generation of surgeon scientists and clinical trialists. This can only come through efforts that creates a culture supportive of scientific discovery [10].

## Efficacy of surgical education

The formula emphasizes the importance of surgical research to obtain the best knowledge for delivery of clinical care. Education is the vector that brings this knowledge to the clinical surgeon or team. There are several examples of deviation between published guidelines and the actual delivery of routine surgical care at all levels [11–13]. A large variation in adoption of trial results into clinical practice is reported, with clinical change taking up to a decade and longer to occur [14]. Thus, availability of improved data-driven knowledge may be slow to impact on clinical practice, despite being contrary to advise given in published guidelines [11]. Such failure to adopt and apply best clinical practice may lead to worse outcomes and to higher costs of unnecessary procedures and complications [15].

In educating the surgeon, the surgeon team members and the patients, novel tools should be incorporated to reach the intended audience with information and new knowledge to increase compliance to best practice. Modern didactic methods such as flipped classroom, team-based learning, social media, and gamification demonstrate increased engagement and may be improve educational efficacy. Finding the most efficient mode of educating trainees, reaching experienced surgeons and involve and inform patients is essential to enhance knowledge distribution. Furthermore, as far as possible, surgical education should happen in surgeons’ own home ground. For training of individuals and teams, there is a strong need to incorporate this training into the daily routine, then being done in a familiar environment. Examples include implementation of structured feedback during training for laparoscopic appendectomy [16] or the use of structured team training in trauma care [17].

## Local implementation

Delivering high-quality care is paramount to patient outcomes. Only through effective implementation strategies can the clinical practice then change for improved care. However, evaluations of implementation of quality improvement initiatives often show mixed results [18]. Notably, this may be due to a lack of training of surgeons on how to implement quality improvements [19]. There is no gain in outcomes from randomized trials if the trials results and lessons are not put into clinical practice. Similar, there is no gain unless best practice of care or best knowledge for training is not incorporated into routine practice. New knowledge that documents improved care needs to be implemented outside the environment where this knowledge emerged. Unfortunately, data suggest that few RCTs have immediate impact on surgical practice [20]. This may be the result of the RCTs per se if perceived as not being generalizable in design or inconclusive in results. There is still a huge variation in practice even for simple measures such as skin prepping before surgery [21] and use of checklists prior to surgery [22] despite strong association with post-operative outcomes. The universal presence of an enhanced recovery protocol after surgery was still found to have variation in adherence between clinical units with measured differences in length of stay [23].

Non-compliance to suggested guidelines may come from several barriers. Disagreement with evidence interpretation, patient’s non-compliance, and lack of structural and institutional resources may all contribute to low efficacy. Increased complexity in healthcare may represent barrier to compliance in practice. Furthermore, some caveats to local buy-in include increasing prevalence of burn-out in surgeons, lack of time and resources from the clinic, failure of administrative staff to identify and give support, and low motivation from staff members. “Implementation science” is a poorly understood field in surgery, yet an essential crux to manage local implementation of ideas and leading change.

Several factors may be more likely to impact change on clinical care. It is not enough to conduct good research and to know about the data. The data needs to be implemented through champions of the methods and pioneers of practice.

## Non-technical skills

Non-technical skills have emerged as essential parts to surgical care, team performance and patient safety [24–26]. A major component of surgical practice consists of teamwork. The concept of a ‘team’ is fluid and may take several forms, from managing and conducting ward rounds, to the composition of multidisciplinary teams for complex disease (e.g. cancer or transplantation) to being part of a trauma team or emergency surgery team. Managing the team in the operating room has just recently become attention of focused research and training [25]. Non-technical skills, such as situation awareness, decision making, leadership, communication, and teamwork play a crucial role on the quality of care and patient safety in the operating room. Non-technical skills training improved team dynamics, safer patient care, and empowerment of team members.

In addition, increased focus on work-life balance and mental health issues are brough on the agenda due to high levels of burn out, depression and suicidal ideation among trainees. Bad behaviour and influence on trainee wellbeing and impact on the surgical workforce and patient care [27].

Lastly, opportunities to improve surgical performance have been limited for practicing surgeons. Surgical coaching has emerged as one strategy to address this need, although a uniform definition and structure to the concept is lacking [28]. However, surgeons report high perceived impact of peer coaching on both patient care and surgeons’ well-being. Novel concepts of teaching and learning through preceptoring, proctoring, mentoring, and coaching have emerged as models to address the educational needs of learners at all levels [29]. In brief, the non-technical skills needed to achieve optimal outcomes in surgical care are being embraced as essential, although much needs to be done to give this form and function from early training and into life-long practice.

## Conclusion

This article proposes a formula for survival in surgery that is based on science, education, implementation, and nontechnical skills adaption through a culture that allows these elements to be emphasized, supported, and sustained. The cultural environment in which the clinical practice occurs is instrumental in bringing science, knowledge, and delivery of care in a team-oriented and patient-focused journey of excellent and safe surgical care. The end-effect should be an optimalization of all elements that are needed to achieve the best possible outcomes for the individual patient. The generic formula for survival in surgery may be utilized to drive focused practice improvement for specific patient journeys or surgical procedures, to drive and pursue excellence in surgical training, and to define and improve the specific elements needed to enhance outcome for surgical team members.
